# Structure–activity relationship-based chemical classification of highly imbalanced Tox21 datasets

**DOI:** 10.1186/s13321-020-00468-x

**Published:** 2020-10-27

**Authors:** Gabriel Idakwo, Sundar Thangapandian, Joseph Luttrell, Yan Li, Nan Wang, Zhaoxian Zhou, Huixiao Hong, Bei Yang, Chaoyang Zhang, Ping Gong

**Affiliations:** 1 School of Computing Sciences and Computer Engineering, University of Southern Mississippi, Hattiesburg, MS 39406 USA; 2 Environmental Laboratory, U.S. Army Engineer Research and Development Center, Vicksburg, MS 39180 USA; 3 Bennett Aerospace Inc, Cary, NC 27518 USA; 4 Department of Computer Science, New Jersey City University, Jersey City, NJ 07305 USA; 5 Division of Bioinformatics and Biostatistics, National Centre for Toxicological Research, U.S. Food and Drug Administration, Jefferson, AR 72079 USA; 6 School of Information & Engineering, Zhengzhou University, Zhengzhou, 450000 China

**Keywords:** Structure–activity relationship (SAR), Chemical classification, Molecular fingerprints, Random forest (RF), Ensemble learning, Bootstrap aggregation (bagging), Class distribution imbalance, Resampling, Synthetic minority over-sampling technique (SMOTE), Edited nearest neighbor (ENN), Random undersampling (RUS)

## Abstract

The specificity of toxicant-target biomolecule interactions lends to the very imbalanced nature of many toxicity datasets, causing poor performance in Structure–Activity Relationship (SAR)-based chemical classification. Undersampling and oversampling are representative techniques for handling such an imbalance challenge. However, removing inactive chemical compound instances from the majority class using an undersampling technique can result in information loss, whereas increasing active toxicant instances in the minority class by interpolation tends to introduce artificial minority instances that often cross into the majority class space, giving rise to class overlapping and a higher false prediction rate. In this study, in order to improve the prediction accuracy of imbalanced learning, we employed SMOTEENN, a combination of Synthetic Minority Over-sampling Technique (SMOTE) and Edited Nearest Neighbor (ENN) algorithms, to oversample the minority class by creating synthetic samples, followed by cleaning the mislabeled instances. We chose the highly imbalanced Tox21 dataset, which consisted of 12 in vitro bioassays for > 10,000 chemicals that were distributed unevenly between binary classes. With Random Forest (RF) as the base classifier and bagging as the ensemble strategy, we applied four hybrid learning methods, i.e., RF without imbalance handling (RF), RF with Random Undersampling (RUS), RF with SMOTE (SMO), and RF with SMOTEENN (SMN). The performance of the four learning methods was compared using nine evaluation metrics, among which F_1_ score, Matthews correlation coefficient and Brier score provided a more consistent assessment of the overall performance across the 12 datasets. The Friedman’s aligned ranks test and the subsequent Bergmann-Hommel post hoc test showed that SMN significantly outperformed the other three methods. We also found that a strong negative correlation existed between the prediction accuracy and the imbalance ratio (IR), which is defined as the number of inactive compounds divided by the number of active compounds. SMN became less effective when IR exceeded a certain threshold (e.g., > 28). The ability to separate the few active compounds from the vast amounts of inactive ones is of great importance in computational toxicology. This work demonstrates that the performance of SAR-based, imbalanced chemical toxicity classification can be significantly improved through the use of data rebalancing.

## Introduction

Structure–activity relationship (SAR) has been frequently used to predict the biological activities of chemicals from their molecular structures. One of the major challenges in SAR-based chemical classification or drug discovery is the extreme imbalance between active and inactive chemicals [1]. Despite the existence of as many as 10^7^ commercially available molecules [2], there is almost always a skew in the distribution of molecules across the bioactivity landscape or toxicity classes. Biomacromolecules such as proteins are often highly selective in their binding to small molecular ligands. Regardless of the huge chemical space, only a few compounds are likely to interact with a target biomacromolecule causing biological effects and are consequently labelled as active compounds, whereas the remaining majority are labelled as inactive compounds. This gives rise to a common problem of class imbalance for SAR-based predictive modeling, particularly in chemical classification and activity quantification using machine learning approaches [3–5].

In machine learning, classifiers are built on data statistics and require a balanced data distribution to achieve optimal performance. Classifiers trained from imbalanced data tend to have a bias towards the majority class. This leads to low sensitivity and precision for the minority class [6], even though the minority class is usually of greater importance than the majority class [7, 8]. In fields such as toxicology and disease diagnosis, bias towards the majority class may result in a higher rate of false negative predictions [1].

The problem of data imbalance has been studied in the context of machine learning-based SAR modeling for more than two decades [7, 9, 10]. As a result, a plethora of methods have been proposed to alleviate the skewness of class distribution. These methods can be grouped into three categories: data-level, algorithm-level, and hybrid [7, 11]. Data-level methods aim to rebalance the training dataset’s class distribution either by undersampling the majority class or oversampling the minority class [12, 13]. They also include methods that clean overlapping samples and remove noisy samples that may negatively affect classifiers [13, 14]. Algorithm-level methods attempt to alter a given learning algorithm by inducing cost sensitivity that biases a model towards the minority class. For example, this may be achieved by imposing a high misclassification cost for the minority class [7, 11]. Recently, Mondrian conformal prediction (MCP) has been applied to improve the performance of machine learning from imbalanced datasets by computing nonconformity scores to model the reliability of predictions. This allows for identifying reliable predications at user-defined significance and confidence levels [15–19]. The MCP approach does not require data rebalancing. Hybrid methods combine the use of resampling strategies with special-purpose learning algorithms [11]. Ensemble approaches (e.g., bagging and boosting), known to increase the accuracy of single classifiers, have also been hybridized with resampling strategies [6].

The selection of appropriate metrics plays a key role in evaluating the performance of imbalanced learning algorithms [11, 20]. In consideration of user preference (e.g., identifying rare active chemicals) and data distribution, a number of metrics have been proposed, including precision, recall, Area Under the Precision-Recall Curve (AUPRC) [21], Area Under the Receiver Operating Characteristics (AUROC) [22], F-measure, geometric mean (G-mean), balanced accuracy, etc. [23–26]. For instance, precision is not affected by a large number of negative samples because it measures the number of true positives out of the samples predicted as positives (i.e., true positive + false positive). A high AUPRC represents both high recall and high precision. High precision relates to a low false positive rate, and high recall relates to a low false negative rate [21, 27].

The present study was motivated by the scarcity of reported efforts in the application of the above-mentioned methods to the SAR-based chemical classification domain. We conducted a literature survey which only identified a few studies in this domain where cost-sensitive learning [28, 29], resampling [29, 30], conformal prediction [18] and extreme entropy machines [1, 31] were employed to specifically deal with data imbalance. Although predictive modeling was improved for certain datasets, a consistent performance enhancement was not observed as a result of resampling and algorithm modification. Apparently, more studies are warranted to further examine such questions as: (1) Does imbalance ratio (IR), i.e., inactive-to-active sample ratio, affect the effectiveness of data-level methods (particularly resampling methods)? (2) Would different data rebalancing techniques affect the performance of a classifier differentially, and does the combination of undersampling and oversampling techniques, such as SMOTEENN (SMOTE + ENN) [32], outperform an undersampling or oversampling technique alone? (3) What metrics can better evaluate the results of imbalanced learning in SAR-based chemical classification? This study attempted to address all three of these questions.

To address the first question, we selected twelve binary datasets of 10 K compounds with varying degrees of imbalance, which were generated within the Toxicology in the 21st century (Tox21) program [33] and used for the Tox21 Data Challenge 2014 [34, 35] (https://tripod.nih.gov/tox21/challenge/about.jsp). To address the other two questions, we chose nine evaluation metrics, compared three resampling algorithms integrated with the base classifier (random forest—RF), and performed statistical analysis to rank the metrics.

In this work, we selected RF as the base classifier and bagging as the ensemble learning algorithm to improve the stability and accuracy of model predictions. Then, we applied three representative resampling methods for data imbalance handling, i.e., random under-sampling (RUS), the synthetic minority over-sampling technique (SMOTE) and SMOTEENN (i.e., a combination of SMOTE and Edited Nearest Neighbor (ENN) algorithms). Consequently, four hybrid learning methods, i.e., RF without imbalance handling (RF), RF with RUS (RUS), RF with SMOTE (SMO), and RF with SMOTEENN (SMN) were tested. Here, we did not intend to conduct a comprehensive or exhaustive comparative investigation of all existing imbalance handling methods, but rather to use this case study to demonstrate that appropriate handling of imbalanced data and the choice of appropriate evaluation metrics could improve SAR-based classification modelling. We also investigated the performance of these existing approaches and highlighted their limitations regarding imbalance ratio. The rest of the paper is organized as follows: “Materials and methods” section covers the study design, data curation and preprocessing steps, imbalance handling methods, and performance metrics. “Results and discussion” section presents our classification performance results, statistical analysis, and a comparison with published results for the Tox21 datasets. Lastly, “Conclusions” section briefly summarizes the major findings from this study and concludes with some remarks on future research needs.

## Materials and methods

### Study design

**Fig. 1:**
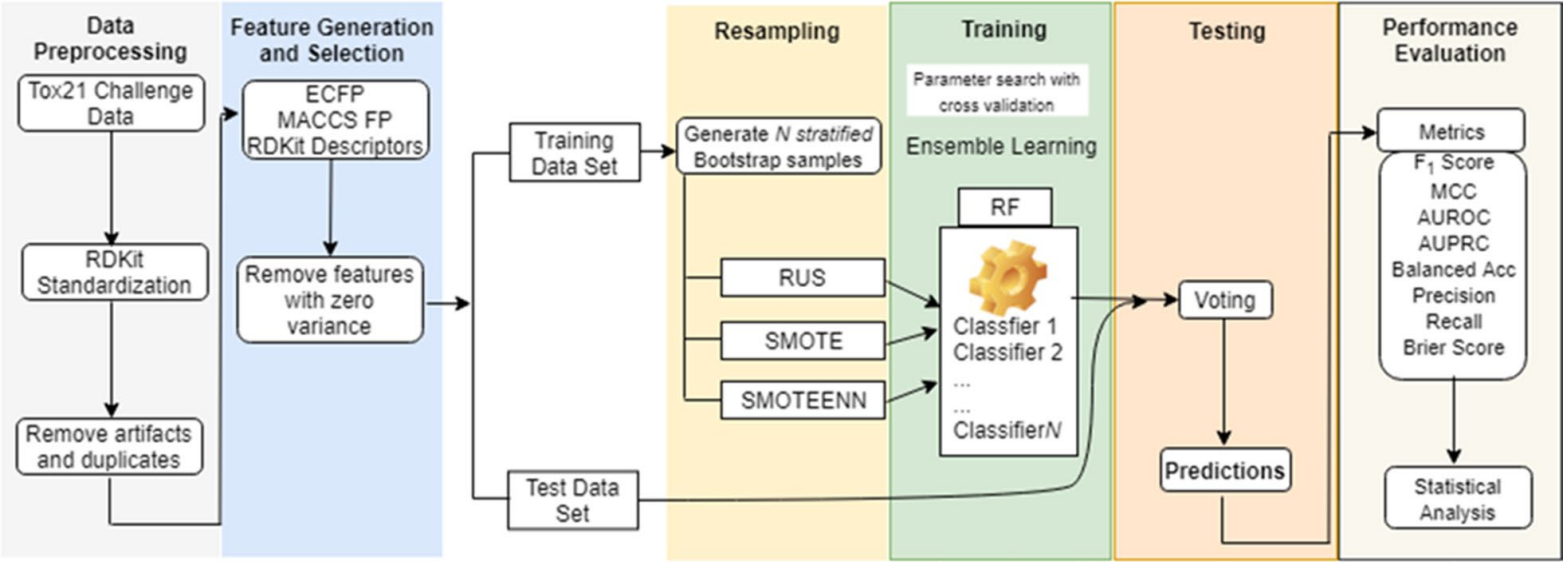
Workflow of structure–activity relationship (SAR)-based chemical classification with imbalanced data processing designed for this study

The workflow of our study design is outlined in Fig. 1. It consists of data preprocessing, feature generation and selection, resampling, model training (ensemble learning), model testing and performance evaluation. The data preprocessing and feature generation steps were applied to a total of 12,707 compounds in the raw dataset of 12 assays. However, feature selection, resampling and training of classifiers were conducted separately for each individual assay. For each assay, the preprocessed compounds in the training set were split into *N* stratified bootstrap samples with replacement (i.e., samples were randomly selected but retained the same imbalance ratio). This was followed by ensemble learning either without resampling (RF) or with the application of a resampling technique (RUS, SMOTE, or SMOTEENN). Optimal parameters for each base learner were obtained via grid search with fivefold cross validation. Optimized base learners were combined to form the final ensemble learner. Evaluation metrics were calculated using the prediction results of RF, RUS, SMO and SMN to statistically compare their performance. Details of the workflow are presented below.

### Chemical in vitro toxicity data curation

The Tox21 Data Challenge dataset used in this study consisted of 12 quantitative high throughput screening (qHTS) assays for a collection of over 10 K compounds (with redundancy within and across assays). The 12 in vitro assays included a nuclear receptor (NR) signaling panel and a stress response (SR) panel. The NR panel comprised 7 qHTS assays for identifying compounds that either inhibited aromatase or activated androgen receptor (AR), aryl hydrocarbon receptor (AhR), estrogen receptor (ER), or peroxisome proliferator-activated receptor γ (PPAR-γ). The SR panel contained 5 qHTS assays for detecting agonists of antioxidant response element (ARE), heat shock factor response element (HSE) or p53 signaling pathways, disruptors of the mitochondrial membrane potential (MMP), or genotoxicity inducers in human embryonic kidney cells expressing luciferase-tagged ATAD5. There were three sets of chemicals: a training set of 11,764 chemicals, a leaderboard set of 296 chemicals and a test set of 647 chemicals [35]. For this study, we merged the leaderboard set with the original training set to form our “training set” and retained the original test set as our “test set”. The Tox21 dataset was downloaded in SDF format at https://tripod.nih.gov/tox21/challenge/data.jsp. There were four possible assay outcomes for each compound: active, inactive, inconclusive or not tested. Only those chemicals labeled as either active (1) or inactive (0) were retained for this study.

### Compound preprocessing and chemical descriptor (feature) generation

Chemical structures were also downloaded at https://tripod.nih.gov/tox21/challenge/data.jsp as SMILES files. Data standardization/cleaning was carried out using MolVS [36], a publicly available tool built on RDKit [37]. Standardization involved a fragmentation step as described in [25] where compounds possessing distinct structures not linked by covalent bonds were split into separate “compound fragments”. Then, solvent fragments, salts and problematic molecules with inconsistent resonance structures and tautomers [38], which should not contribute to the biological effect of a compound [39], were removed. The resulting SMILES entries were canonicalized by standardizing chemotypes such as nitro groups and aromatic rings, and the largest uncharged fragments of the compound were retained. After standardization, the resulting fragments were merged based on their reported activity to exclude replicates and conflicting instances. Specifically, only one instance of a set of duplicates was retained with the most frequent activity label, while duplicates with ambiguous activity labels (i.e., an equal number of active and inactive outcomes for the same chemical) were removed. Three types of molecular features (> 2000 in total), i.e., RDKit descriptors, MACCS (Molecular ACCess System) keys and Extended-Connectivity Fingerprints (ECFPs) [40] with a radius of 2 and a fixed bit length of 1024, were generated using RDKit [37] to characterize the final set of compounds. All features with zero variance were dropped.

### Sampling and classification methods

Here we briefly describe the three resampling techniques (i.e., RUS, SMOTE and SMOTEENN) that we used for handling imbalanced data with RF chosen as the base classifier.

#### RUS

RUS is a widely used undersampling technique which randomly removes samples from the majority class. In our study, RUS was used to randomly remove inactive compounds. While RUS alleviates imbalance in the dataset, it may potentially discard useful or important samples and increase the variance of the classifier. Recent studies have shown that the integration of RUS with ensemble learning can achieve better results [6, 41]. To overcome its drawbacks, we combined RUS with bagging (an ensemble learning algorithm) for SAR-based chemical classification.

#### SMOTE

SMOTE is an oversampling technique that creates synthetic samples based on feature space similarities between existing examples in the minority class [12]. It has shown a great deal of success in various applications [20]. To create a synthetic data sample, we first took a sample from the dataset of the minority class and considered its *k*-nearest neighbors based on Euclidian distance to form a vector between the current data point and one of those *k* neighbors. The new synthetic data sample was obtained by multiplying this vector by a random number *α* between 0 and 1 and adding the product to the current data point. More technical details on how to create synthetic samples are described in the Additional file 1: Figure S1 and in [12, 20]. Applying SMOTE to the minority class instances can balance class distributions [12] and augment the original dataset in a manner that generally significantly improves learning [20].

#### SMOTEENN

Despite many promising benefits, the SMOTE algorithm also has its drawbacks, including over generalization and variance [20]. In many cases, class boundaries are not well defined since some synthetic minority class instances may cross over to appear in the majority class space, especially for nonlinear data with a large feature space [42]. As a result, some new synthetic samples in the minority class may be mislabeled and attempting to learn from such datasets often results in a higher false prediction rate [43]. To remove the mislabeled samples created by the SMOTE technique, we applied SMOTEENN [32], a combination of SMOTE and the Edited Nearest Neighbor (ENN) [44] algorithm, to clean the synthetic data samples.

In the ENN algorithm, the label of every synthetic instance is compared with the vote of its *k*-nearest neighbors. The instance is removed if it is inconsistent with its *k*-nearest neighbors; otherwise, it remains in the dataset. The process of removing mislabeled samples and retaining the valid synthetic instances is illustrated in the Additional file 1: Figure S1c. A higher *k* value in the edited nearest neighbors algorithm leads to a more stringent cleaning rule that allows more synthetic instances to be eliminated. Applying SMOTEENN to an imbalanced dataset does not automatically result in a perfectly balanced set after resampling, but it creates more meaningful synthetic samples in the minority class and reduces the imbalance ratio to a more manageable level.

##### RF and ensemble learning

RF is a robust supervised learning algorithm that has been widely used for classification in many applications in data science [45]. An RF model consists of many individual decision trees that operate as an ensemble. The individual decision trees are generated using a random selection of features at each node to determine the split. During classification, each tree votes and the class with most votes becomes the model’s prediction.

RF can be built [46] and improved [47] using bagging (short for bootstrap aggregation). Bagging is a common ensemble method that uses bootstrap sampling in which several base classifiers are combined (usually by averaging) to form a more stable aggregate classifier [48]. Each base classifier (RF in this study) in the ensemble is trained on a different subset of the training dataset obtained by random selection with replacement, thus introducing some level of diversity and robustness. It is well known that the bagging classifier is more robust in overcoming the effects of noisy data and overfitting, and it often has greater accuracy than a single classifier because the ensemble model reduces the effect of the variance of individual classifiers [6, 48, 49].

In our case, the Tox21 dataset was both highly dimensional and highly imbalanced [6, 50]. For datasets with such a large feature space and a small number of minority class samples, classification often suffers from overfitting. Because bagging is less susceptible to model overfitting, we chose it as the ensemble method. Combining the base classifier RF with three sampling techniques (RUS, SMO and SMOTEENN) and bagging, we assembled four hybrid classification methods: (1) RF without resampling, (2) RF + RUS, (3) RF + SMO, and (4) RF + SMOTEENN. For more convenient result analysis, the four methods were simply denoted as RF, RUS, SMO and SMN, respectively.

Here we use SMN as an example to illustrate the algorithm that integrates resampling with ensemble learning (see Algorithm 1 and Fig. 1). First, a subset, ${S}_{i}$, was obtained by taking a stratified bootstrap sampling from the training set, $X$. This sampling process was repeated *N* times, where *i* = 1 to *N,* with *N* ranging between 5 and 100 in steps of 5. Stratification was employed to ensure that each bootstrap had the same class distribution as the entire training set. Each subset is used to train a classifier in the ensemble, hence *N* is also equivalent to the number of classifiers. Then, the SMOTEENN algorithm was applied to ${S}_{i}$ to oversample the minority class and obtain an augmented training subset $S^{\prime}_{i}$, which was used to train a random forest classifier ${f}_{i}\left(x\right)$. The parameters for each classifier in the ensemble were selected using a grid search with a fivefold cross-validation. This would give every individual classifier a chance to attain its best performance and contribute optimally to the ensemble. The final ensemble model was a bagged classifier that would count the votes of the *N* classifiers and assign the class with the most votes to a chemical in the test dataset. The other three methods RF, RUS and SMO also employed Algorithm 1 with the only difference being the resampling technique, i.e., no resampling, RUS and SMOTE, respectively. All classifiers were implemented using the Scikit-learn package [51] and Imbalanced-learn in a Python toolbox [52].
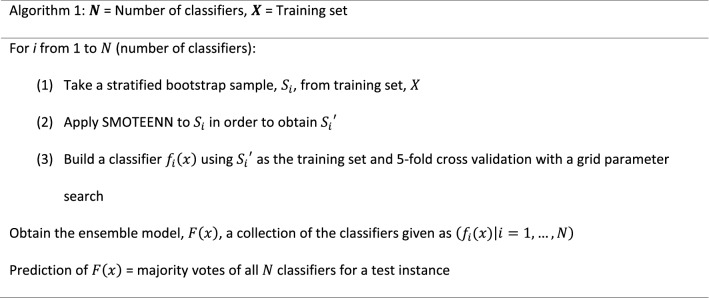


### Performance evaluation metrics

The output of a binary classification model can be primarily represented by four terms: (1) true positive (TP) defined as the number of true active chemicals that are correctly predicted as active by the model; (2) false positive (FP) as the number of true inactive chemicals incorrectly predicted as active; (3) true negative (TN) as the number of true inactive chemicals correctly predicted as inactive; and (4) false negative (FN) as the number of true active chemicals incorrectly predicted as inactive. Most evaluation metrics are derived from these four terms. True positive rate (TPR), also referred to as sensitivity or recall, represents the fraction of correctly predicted active chemicals. In SAR modeling, recall is also considered as a measure of the accuracy of the active (minority) class. True negative rate (TNR) or specificity provides a similar measure (accuracy) for the inactive (majority) class. Precision estimates the probability of a model to make a correct active class prediction. F_1_ score is the harmonic mean of precision and recall. Similarly, balanced accuracy (BA) is the average of correct predictions for both classes. Matthews correlation coefficient (MCC) offers a good index for the performance of imbalanced classification tasks as it incorporates all the components of the confusion matrix [53]. MCC has been widely used to evaluate the performance of SAR-based chemical classification [34, 54]. The MCC value varies in the range of [− 1, 1] with − 1 implying disagreement, 1 complete agreement and 0 no correlation between the prediction and the known truth. The Brier score is a measure of the average squared difference between the predicted probabilities and the known value for a class, and it assesses the overall accuracy of a probability model. The formulas of these evaluation metrics are given as follows:

$${\text{Recall}} = {\text{Sensitivity}} = \frac{TP}{{TP + FN}}$$

$${\text{Specificity}} = \frac{TN}{{TN + FP}}$$

$${\text{Precision}} = \frac{TP}{{TP + FP}}$$

$${\text{F}}_{{1}} \,{\text{score}} = 2 \times \frac{Precision \times Recall}{{Precision + Recall}}$$

$${\text{Balanced accuracy }}\left( {{\text{BA}}} \right) = \frac{Sensitivity + Specificity}{2}$$

$${\text{MCC}} = \frac{{TP \times TN{ }{-}{ }FP \times FN}}{{\sqrt {\left( {TP + FP} \right)\left( {TP + FN} \right)\left( {TN + FP} \right)\left( {TN + FN} \right)} }}$$

$${\text{Brier score}} = \frac{1}{N}\sum\nolimits_{i = 1}^{N} {\left( {p_{i} - o_{i} } \right)}^{2}$$

where *N* is the total number of chemicals in a dataset, ${p}_{i}$ ($\in \left[\mathrm{0,1}\right])$ is the predicted probability, and ${o}_{i}$ is the ground truth for the *i*th chemical (equal to 1 for active and 0 for inactive).

In addition, the two widely used metrics AUROC and AUPRC were also calculated using Scikit-learn [51] to evaluate and compare the overall performance of a classifier against another. Finally, sensitivity–specificity gap (SSG), calculated as the absolute value of the difference between sensitivity and specificity, was introduced as a metric to evaluate how balanced a classifier was in terms of its performance on these two metrics [13].

We performed statistical analysis to assess if there existed significant differences among the four investigated classification methods in terms of their performance metrics across the twelve bioassays (Table 1). We adopted a nonparametric test for multiple comparisons as described in Garcia et al. [55]. Using the Statistical Comparison of Multiple Algorithms in Multiple Problems (scmamp) library in R [56], we conducted a Friedman’s aligned-rank test [57]. The Friedman test was chosen over other statistical tests such as ANOVA because it does not require the assumption of data normality. The Bergmann-Hommel post-hoc test was carried out for pairwise comparisons between SMN and the other three methods (RF, RUS and SMO) [54].

**Table 1:** Class distribution and imbalance ratio (IR) of the preprocessed training and test chemical datasets from Tox21 Data Challenge

In vitro qHTS assay ID	Total number of chemicals	Training set			Test set		
		Inactive	Active	IR	Inactive	Active	IR
NR-AR	6436	5698	166	34.3	560	12	46.7
NR-AR-LBD	5931	5223	143	36.5	557	8	**69.6**
NR-AhR	5596	4445	561	7.9	520	70	7.4
NR-Aromatase	4901	4193	193	21.7	478	37	12.9
NR-ER	5171	4167	500	8.3	455	49	9.3
NR-ER-LBD	6043	5239	221	23.7	563	20	28.2
NR-PPAR-γ	5712	5005	120	**41.7**	558	29	19.2
SR-ARE	4808	3669	603	6.1	448	88	**5.1**
SR-ATAD5	6320	5515	203	27.2	568	34	16.7
SR-HSE	5529	4733	206	23.0	573	17	33.7
SR-MMP	4955	3763	666	**5.7**	472	54	8.7
SR-p53	6009	5110	303	16.9	558	38	14.7

The highest and lowest IRs for the training and test sets are in bold

## Results and discussion

In this section, we present (1) a summary of the curated and preprocessed Tox21 dataset, (2) the preliminary comparative results to justify the selection of RF as the base classifier, (3) parameter optimization for RF and ENN algorithms, (4) performance metrics of four classification methods for the twelve imbalanced Tox21 datasets, (5) the impact of IR and classification methods on prediction performance, and (6) a comparison between this study and published Tox21 studies.

### Data curation and preprocessing

A summary of the preprocessed training and test datasets of chemicals and their activities measured by 12 qHTS in vitro assays is presented in Table 1. Although the original raw Tox21 datasets contained more than 12 K chemicals, approximately 50% of them or fewer were retained for each assay after preprocessing. This was primarily due to duplication and the absence of testing data for individual assays. The imbalanced ratio (IR), defined as the ratio of the number of the majority class (inactive compounds) to that of the minority class (active compounds) [42], varied widely between assays and between the training and the test sets. Such large disparities offered a great opportunity to investigate the performance of different ensemble-resampling approaches as a function of IR (see below for detailed results). In the training datasets, the highest IR of 41.7 appeared in the dataset of the NR-PPAR-γ assay, whereas the lowest IR of 5.7 was observed with the SR-MMP assay. The test datasets generally had IRs larger than or equivalent to those of their corresponding training datasets, e.g., measuring as high as ~ 70 for NR-AR-LBD (except for NR-Aromatase, NR-PPAR-γ, and SR-ATAD5).

### Selecting RF as the base classifier

**Fig. 2:**
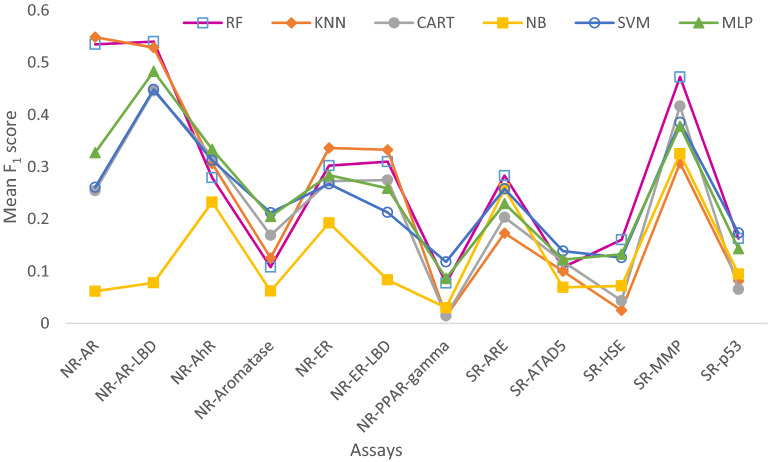
A spot check of six popular machine learning algorithms: performance of classifiers trained using the preprocessed Tox21 training datasets as evaluated using F_1_ score

A comparison of six popular machine learning algorithms, i.e., RF, K-nearest neighbors (KNN), decision trees (CART), Naïve Bayes (NB), support vector machine (SVM) and multilayer perceptron (MLP), was performed using the training datasets of all twelve assays and a stratified fivefold cross validation. These algorithms were all implemented in Scikit-learn [51] with default parameter settings. The purpose of this preliminary study was to select a base classifier from these algorithms. F_1_ score was calculated and used as the metric to evaluate classification performance. As shown in Fig. 2, RF was the frontrunner for four of the 12 assay datasets, including NR-AR-LBD, SR-ARE, SR-HSE, and SR-MMP. RF was the second best performer for another five assays (i.e., NR-AR, NR-ER, NR-ER-LBD, NR-PPAR-γ, and SR-p53). The average F_1_ score of RF for all 12 assays was the highest (0.2783) among all six algorithms, and the runner-up was MLP with an average F_1_ score of 0.2487. Clearly, RF outperformed the other five algorithms on the Tox21 dataset, which informed our decision to proceed with choosing RF as the base classifier and to focus our study on imbalance handling methods.

Furthermore, the RF classifier was widely used by the participating teams in the Tox21 Data Challenge [28, 48]. Two of the winning teams developed RF models that achieved the best performance in predicting compound activities against AR, aromatase, and p53 [58] as well as ER-LBD [59]. Using the same RF classifier and the same dataset made it convenient to compare our results with those from the participating teams and allowed us to better investigate the impact of resampling methods on improving imbalanced learning and, consequently, improving classification performance (see “Comparison with Tox21 Data Challenge winners” section below for more info).

### Parameter optimization for the RF classifier

**Fig. 3:**
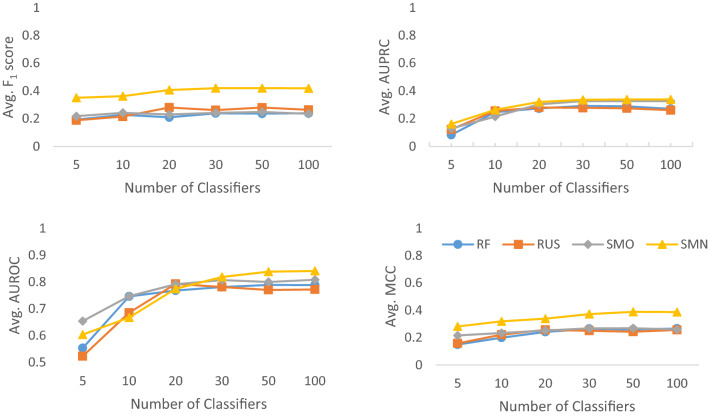
The relationship between model performance and the number of classifiers in the RF base classifier

It is generally accepted that the accuracy of a classifier ensemble is positively correlated with ensemble diversity [60]. Here, we adjusted the ensemble diversity by randomly selecting data instances to create the bootstrap samples (see Fig. 1) and by increasing the number of classifiers included in the ensemble. Figure 3 shows that the performance of classifier ensembles measured by the average F_1_ score, AUPRC, AUROC and MCC for all four methods changes with the varying number of classifiers in the ensemble. A plateau was encountered when the number of classifiers reached 30, which may have been the optimal number of classifiers in this situation. After this point, there was little improvement in performance as the number of classifiers increased. Even if minor improvements were noticed using 100 classifiers for some metrics (e.g., MCC), this dramatically increased the computational time and resources needed to train the model. The relationship between performance and the number of classifiers may be explained by the importance of diversity in ensemble learning. With every bootstrap sample being different from another in terms of chemical composition and fingerprint features, diversity in the bagging ensemble was inherent. However, as the number of classifiers increased, the number of times (frequency) that a sample was selected from the same population also increased. This would result in a decline in the variance between such bootstrap samples or a flat line in ensemble diversity. Consequently, a flat line was observed in performance metrics as the number of classifiers in an ensemble increased from 30 to 100 (Fig. 3). In the subsequent experiments, we adopted the optimal number of 30 classifiers for ensemble learning.

### Optimal number of nearest neighbors (*k*) in the ENN algorithm of SMN models

**Fig. 4:**
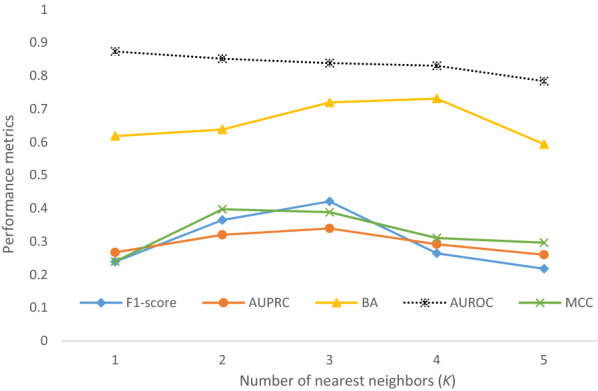
Performance metrics of SMN models measured as the number of nearest neighbors (*k*) varied in the ENN

Another parameter we optimized was the *k* value in the ENN algorithm. The choice of a synthetic instance to be removed from the training set is determined by the voting of its *k* neighbors. As shown in Fig. 4, we varied the number of nearest neighbors *k* from 1 to 5, and 3 appeared to be the optimal *k* value for most of the five measured performance metrics. F_1_ score and AUPRC peaked at *k* = 3, BA plateaued when *k* = 3 or 4, whereas MCC peaked earlier at *k* = 2. AUROC was the only metric not affected by the change in *k* value. Thus, the *k* value was set at 3 for SMN in this study.

By setting *k* at this optimal value, ENN may help increase the classifier’s generalizability by removing noisy (mislabeled) synthetic instances introduced in the SMOTE step. By reducing the amount of noise in the dataset while reducing imbalance, it is expected that the class boundaries between active and inactive compounds can be better defined. A reduction in noisy instances can also reduce the chance of over-fitting. This is essentially where the power of SMN lies. However, further increments in the *k* value beyond the optimum led to a decline in classifier performance.

### Performance evaluation metrics

Table 2 reports nine performance metrics and their average values for four classification methods (RF, RUS, SMO and SMN) for 12 bioactivity assays, with the best performer highlighted in bold for each evaluation metric and assay. The derived specificity results are reported alone with sensitivity and SSG results in Additional file 1: Table S1. For each assay, the training dataset was employed to train a classifier using four different algorithms, and then the trained classifier was applied to the test dataset to determine performance metrics as described in the “Materials and methods” section (also see Fig. 1). The reported values varied greatly depending on metrics, assays and algorithms. For instance, AUROC has the highest values averaged at 0.8049, whereas MCC has the lowest mean value of 0.2945. This is not surprising as different metrics measure different aspects of learning algorithm performance and trained model quality [61].

**Table 2:** Nine metrics for evaluating the performance of four classification methods (RF, RUS, SMO and SMN) with twelve Tox21 qHTS assay datasets

Metrics	Classifier	NR-AR	NR-AR-LBD	NR-AhR	NR-Aromatase	NR-ER	NR-ER-LBD	NR-PPAR-γ	SR-ARE	SR-ATAD5	SR-HSE	SR-MMP	SR-p53	Mean	CV^a^ (%)
F_1_ score	RF	0.1538	0.0000	0.4340	0.2326	0.2727	0.2400	0.0606	0.3359	0.2500	**0.2500**	0.5106	0.1364	0.2397	60
	RUS	0.1176	**0.1667**	0.4507	0.2222	0.2605	0.1849	**0.4051**	0.4185	0.2063	0.1058	**0.5867**	0.2527	0.2815	53
	SMO	**0.2500**	0.0000	0.3883	0.1905	0.3692	0.2857	0.1765	0.2927	0.2439	0.1905	0.3902	0.1395	0.2431	47
	SMN	0.1951	0.1111	**0.5856**	**0.5070**	**0.6078**	**0.3636**	0.3929	**0.6791**	**0.3636**	0.2400	0.5850	**0.4225**	**0.4211**	**42**
MCC	RF	**0.2859**	− 0.0050	0.4101	0.3202	0.2726	0.2891	0.0767	0.2770	**0.3377**	**0.2619**	0.4701	0.1801	0.2647	49
	RUS	0.1056	**0.1602**	0.4209	0.1914	0.1816	0.1908	**0.3810**	0.2950	0.2049	0.1190	**0.5537**	0.2769	0.2568	53
	SMO	0.2805	− 0.0071	0.3669	0.2792	0.3990	0.3018	0.2355	0.2498	0.3091	0.2327	0.3662	0.2019	0.2679	**39**
	SMN	0.1886	0.0975	**0.5342**	**0.4711**	**0.5643**	**0.3404**	0.3627	**0.6177**	0.3261	0.2226	0.5492	**0.3872**	**0.3885**	42
AUROC	RF	**0.8232**	0.7963	0.9063	0.7356	0.7601	0.6963	0.6640	0.7867	0.7827	0.7610	0.9194	0.7443	0.7813	10
	RUS	0.6785	**0.9133**	0.8852	0.7627	0.7174	0.7619	**0.7937**	0.7698	0.7791	0.7065	0.9295	0.8168	0.7929	10
	SMO	0.7780	0.7509	0.8936	0.8112	0.7296	0.8072	0.7872	0.7714	**0.8151**	0.7983	0.8893	0.8510	0.8069	**6**
	SMN	0.6810	0.7969	**0.9196**	**0.8500**	**0.8628**	**0.8233**	0.7713	**0.8910**	0.8093	**0.8483**	**0.9294**	**0.8785**	**0.8384**	8
AUPRC	RF	**0.3521**	0.0565	**0.5846**	0.2825	0.3203	0.1887	0.1120	0.4224	0.2881	0.1608	**0.5632**	0.1881	0.2933	57
	RUS	0.1444	**0.1068**	0.4836	0.2043	0.2420	0.1545	**0.5067**	0.4140	0.2423	0.0622	0.5237	0.2295	0.2762	59
	SMO	0.3290	0.0821	0.5065	0.3504	0.3895	**0.2658**	0.2806	0.4052	**0.3350**	**0.1993**	0.4928	0.2913	0.3273	**36**
	SMN	0.0685	0.0639	0.5660	**0.3845**	**0.5688**	0.2018	0.3736	**0.6443**	0.2422	0.1134	0.5234	**0.3254**	**0.3396**	60
Balanced accuracy (BA)	RF	0.5417	0.4991	0.6518	0.5665	0.5830	0.5732	0.5146	0.6016	0.5726	0.5847	0.7053	0.5368	0.5776	10
	RUS	0.5929	**0.6124**	0.8129	0.6828	0.6513	**0.6968**	**0.7454**	0.6977	**0.7133**	**0.6665**	**0.8523**	**0.7777**	0.7085	11
	SMO	0.5815	0.4982	0.6304	0.5530	0.6181	0.5964	0.5499	0.5833	0.5718	0.5571	0.6354	0.5377	0.5761	**7**
	SMN	**0.6443**	0.5544	**0.8228**	**0.7265**	**0.7922**	0.6858	0.6753	**0.8545**	0.7018	0.6529	0.8452	0.6812	**0.7198**	13
Precision	RF	**1.0000**	0.0000	**0.6389**	**0.8333**	0.5294	**0.6000**	0.2500	0.5116	**0.8333**	0.4286	**0.6000**	0.5000	**0.5604**	48
	RUS	0.0769	0.1250	0.2991	0.1302	0.1604	0.1111	0.3200	0.2869	0.1193	0.0576	0.4583	0.1464	0.1909	64
	SMO	0.5000	0.0000	0.6061	0.8000	**0.7500**	0.5000	**0.6000**	0.5143	0.7143	**0.5000**	0.5714	**0.6000**	0.5547	**36**
	SMN	0.1379	0.1000	0.4775	0.5294	0.5849	0.3333	0.4074	**0.5748**	0.2963	0.1818	0.4624	0.4545	0.3784	44
Recall or Sensitivity	RF	0.0833	0.0000	0.3286	0.1351	0.1837	0.1500	0.0345	0.2500	0.1471	0.1765	0.4444	0.0789	0.1677	75
	RUS	0.2500	**0.2500**	**0.9143**	**0.7568**	**0.6939**	**0.5500**	**0.5517**	0.7727	**0.7647**	**0.6471**	**0.8148**	**0.9211**	**0.6573**	**34**
	SMO	0.1667	0.0000	0.2857	0.1081	0.2449	0.2000	0.1034	0.2045	0.1471	0.1176	0.2963	0.0789	0.1628	54
	SMN	**0.3333**	0.1250	0.7571	0.4865	0.6327	0.4000	0.3793	**0.8295**	0.4706	0.3529	0.7963	0.3947	0.4965	43
Brier score (BS)	RF	**0.3817**	0.5425	0.3404	0.3997	0.3883	0.4163	0.3961	0.3725	0.3947	0.4257	0.3215	0.3810	0.3967	14
	RUS	0.4461	**0.3874**	0.3104	0.3724	0.3793	0.4299	**0.3204**	0.3735	0.3829	0.4871	0.3892	0.3936	0.3894	**13**
	SMO	0.4263	0.6739	0.3281	0.3379	0.4205	0.4067	0.4138	0.3881	0.3924	0.4146	0.3467	0.3814	0.4109	22
	SMN	0.4303	0.4156	**0.2583**	**0.3327**	**0.3134**	**0.3670**	0.3503	**0.2761**	**0.3431**	**0.3491**	**0.2371**	**0.3014**	**0.3312**	18
Sensitivity–specificity gap (SSG)^b^	RF	0.9167	0.9982	0.6464	0.8628	0.7987	0.8464	0.9601	0.7031	0.8511	0.8165	0.5217	0.9157	0.8198	17
	RUS	0.6857	**0.7249**	0.2028	**0.1480**	**0.0851**	**0.2937**	**0.3874**	0.1499	**0.1027**	**0.0388**	**0.0750**	**0.2867**	**0.2651**	87
	SMO	0.8297	0.9964	0.6893	0.8898	0.7463	0.7929	0.8930	0.7576	0.8494	0.8789	0.6783	0.9175	0.8266	**12**
	SMN	**0.6221**	0.8588	**0.1314**	0.4800	0.3189	0.5716	0.5920	**0.0500**	0.4625	0.6000	0.0978	0.5730	0.4465	55
Average^c^	RF	**0.2157**	− 0.0215	0.3297	0.2048	0.1928	0.1638	0.0396	0.2344	0.2184	0.1535	0.3744	0.1187	0.1854	59
	RUS	0.0927	**0.1358**	0.4171	0.2700	0.2714	0.2140	**0.3329**	0.3479	**0.2827**	**0.2043**	0.4728	**0.3045**	0.2788	**39**
	SMO	0.1811	− 0.0385	0.2956	0.2072	0.2593	0.1953	0.1585	0.2084	0.2105	0.1447	0.2907	0.1557	0.1890	46
	SMN	0.1329	0.0638	**0.4748**	**0.3491**	**0.4424**	**0.2455**	0.2689	**0.5294**	0.2671	0.1848	**0.4840**	0.2966	**0.3116**	47

The metrics were calculated using the test datasets (see Table 1). The best performer among the four classifiers is highlighted in bold for each assay and each evaluation metric. The highest value represents the best performer except for Brier score and sensitivity–specificity gap which are the opposite (i.e., the lower the better). See Additional file 1: Table S1 for the specificity values

^a^Coefficient of variation (CV) = standard deviation/mean of 12 assays

^b^SSG = absolute value of (Specificity—Sensitivity)

^c^Average (of 9 metrics) = (F_1_ + MCC + AUROC + AUPRC + BA + Precision + Recall-BS-SSG)/9. The values of BS and SSG are subtracted (instead of added) to the sum because BS and SSG are negatively correlated to model performance

We excluded accuracy (the ratio of correct predictions to the total number of chemicals) and specificity from the metrics panel presented in Table 2 because accuracy may be misleading in evaluating model performance for highly imbalanced classification [22]. Specifically, a high accuracy does not translate into a high capability of the prediction model to correctly predict the rare class, whereas specificity is less relevant since we are more interested in the positive class (active minority). However, the nine chosen metrics in the panel are not necessarily the ideal ones for evaluating the performance of classification with a skewed class distribution. For instance, both AUROC and AUPRC can provide a model-wide evaluation of binary classifiers [27]. Although AUROC, proposed as an alternative to accuracy [22], is unaffected by data skewness [62], it may provide an excessively optimistic view of an algorithm’s performance on highly imbalanced data [21]. AUPRC, on the other hand, is affected by data imbalance [62], but it is a more informative and more realistic measure than AUROC for imbalanced classification [27]. Another example is precision and recall, both of which depend on a threshold selected to determine if a chemical compound is active or inactive. A higher recall may be obtained by setting a lower threshold (increasing the number of TP predictions and decreasing the number of FN predictions), which results in a lower precision (more FP predictions). On the other hand, raising the threshold for labeling active chemicals may benefit precision but hurt recall. Optimizing both precision and recall occurs with a tradeoff, especially with imbalanced data. F_1_ score appears to be a balanced trade-off between precision and recall. Nevertheless, like AUPRC, F_1_ score is also attenuated by data skewness [62]. SSG, a good indicator of balance between sensitivity and specificity [13], may become an inefficient performance metric when both sensitivity and specificity are low. For such applications as predictive toxicology and drug discovery, one may be more interested in improving sensitivity instead of reducing SSG due to the rarity of positive instances. Given the pros and cons of these metrics, it is necessary to use a suite of metrics for performance evaluation. Hence, we calculated the “average” of the nine metrics (Table 2) which may serve as a comprehensive indicator of model performance. However, its formula (e.g., membership composition, weight of each component metric, and normalization method) and applicability still require further investigation.

### Impact of imbalance ratio on performance metrics

The variation in the same performance metrics between different assay datasets is as high as 87% CV (Table 2), suggesting that dataset properties (IR in particular) have a significant impact. Nevertheless, systematic assessment of the impact of IR on prediction accuracy remains a challenging problem. The IRs in our assay datasets varied from 5 to 70 (Table 1). We calculated correlation coefficients (CCs) between log_2_(IR) and the score of five evaluation metrics (Table 3). Except for the CCs between AUROC and RF/RUS/SMO, there exists a significant negative correlation between IR (of the test datasets) and the performance evaluation metrics F_1_ score, MCC, BA, AUPRC, AUROC, and the average of all 9 chosen metrics. This is consistent with earlier reports on the adverse effects of IR on these metrics [62]. The statistically significant positive correlation between IR and SSG suggests that higher IRs would increase SSG, which is also undesirable.

**Table 3:** Correlation coefficients (CCs) between log_2_IR and six performance metrics plus the average of nine metrics in Table 2 for all four classification algorithms

Metrics	Algorithms			
	RF	RUS	SMO	SMN
F_1_ score	− 0.7217	− 0.7394	− 0.6941	− 0.9817
MCC	− 0.5778	− 0.6180	− 0.6419	− 0.9761
BA	− 0.6539	− 0.6274	− 0.6227	− 0.9461
AUPRC	− 0.7034	− 0.7148	− 0.8418	− 0.9628
AUROC	− **0.277**	− **0.1589**	− **0.3713**	− 0.7417
SSG	0.7158	0.7072	0.7006	0.9195
Average	− 0.6536	− 0.8421	− 0.7725	− 0.9822

Insignificant CCs are highlighted in bold and are those whose absolute values are smaller than 0.5760, the critical value at α = 0.05 significance level for the degree of freedom *df* = 10 (i.e., n−2, where n = 12 assays)

**Fig. 5:**
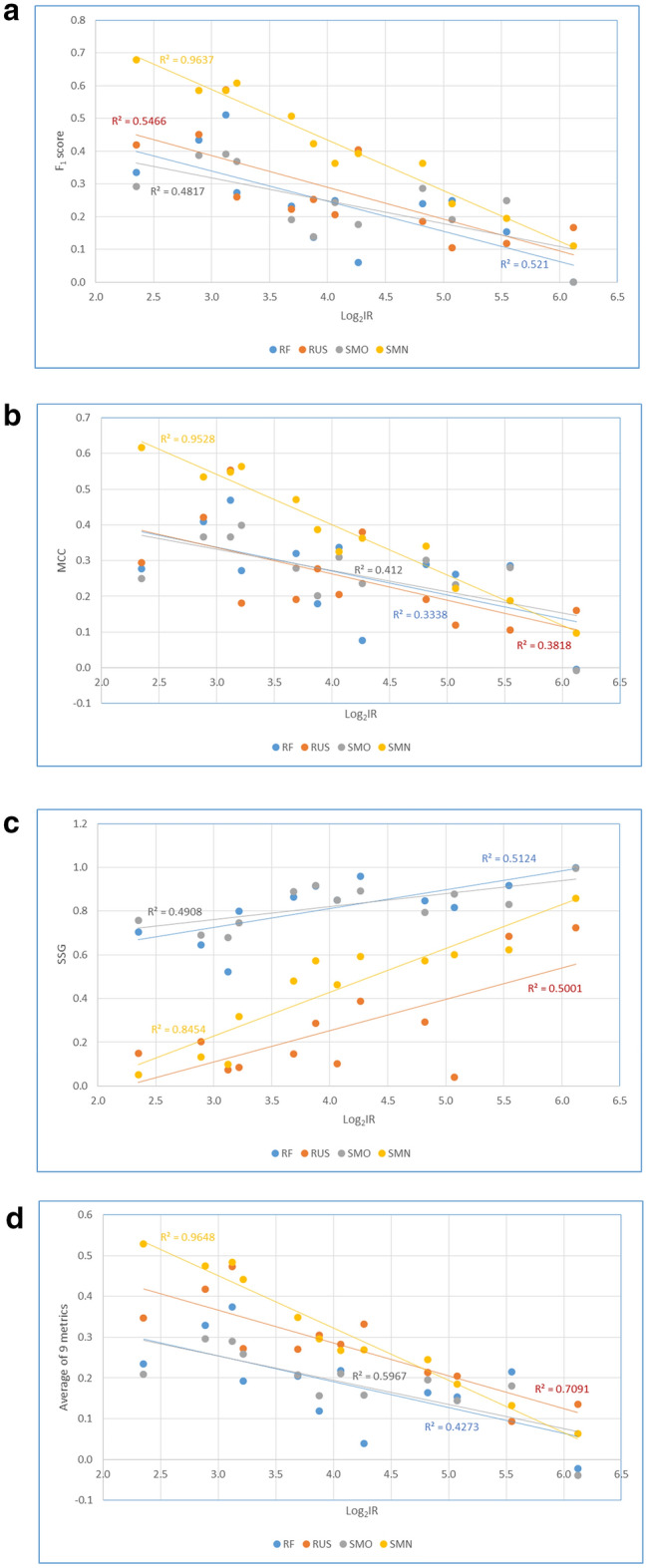
The relationship between imbalance ratio (Log_2_IR) and prediction performance metrics calculated for four classification methods (SMN, SMO, RUS and RF): **a** F_1_ score, **b** MCC, **c** SSG, and **d** the average of 9 metrics

To investigate how IR affects the extent of performance improvement obtained by different resampling techniques, the scores of four metrics (F_1_ score, MCC, SSG and the average of 9 metrics) of all twelve assays are plotted against their log_2_IR (see Fig. 5). For MCC, F_1_ score and the average of 9 metrics, the trend line of SMN is well above those of SMO, RUS and RF, indicating that SMN performed better than other classifiers. The trend lines of SMO and RUS intertwine with that of RF, suggesting that both SMO and RUS did not consistently improve the performance metrics over the base classifier RF. In addition, the SMN trend line intercepts with the other three at about log_2_IR = 4.8 (for average), 5.5 (for MCC) or 6.1 (for F_1_ score), suggesting that a metric-specific IR between 28 and 70 is likely the threshold at which SMN can outperform other classifiers. The lower the IR value is, the more improvements SMN can achieve, compared to the RF, RUS and SMO classifiers. When IR approaches the threshold, the improvements are insignificant. These results demonstrate the limitation of data rebalancing techniques and also provide useful feedback for data acquisition. If evaluated by the SSG metric (the smaller, the better), RUS outperformed SMN and the other two algorithms, suggesting that SMN had limited power in narrowing the gap between sensitivity and specificity. Whenever possible, we should increase the number of active compounds to reduce the imbalance ratio in order to obtain more accurate predictions in SAR-based chemical classification.

### Impact of resampling techniques on classifier performance

**Fig. 6:**
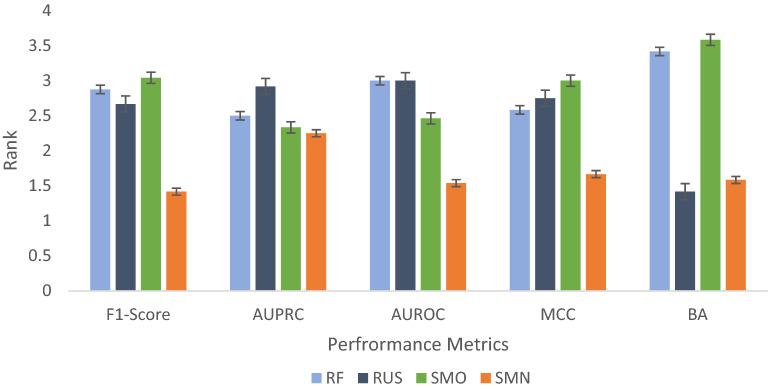
Average Friedman ranks of the four classification methods (RF, RUS, SMO and SMN) with respect to five metrics (F_1_ score, AUPRC, AUROC, MCC and BA). Error bars represent standard errors. See Table 4 for statistical significance in the difference between classifiers

The effect of algorithm choice is partially reflected by a change of 0.1263 in the average metrics score from RF (0.1854) to SMN (0.3116) (Table 2). We also calculated the average Friedman ranking of each classifier [55] by ranking the four algorithms from 1 to 4 based on their performance on each assay dataset. The best classifiers were assigned a rank of 1 and the worst classifiers were assigned a rank of 4. The algorithm with the lowest average rank is considered the best for a specific metric. As shown in Fig. 6, SMN outperformed the other algorithms (RF, RUS and SMO) in terms of four metrics (F_1_ score, AUPRC, AUROC and MCC) and was only slightly surpassed by the frontrunner RUS for the BA metric. Taking F_1_ score as an example, SMN performed better in seven of the 12 assay datasets, followed by RUS which was the best performer for three assays (Table 2). More interestingly, the magnitude of improvement offered by SMN from the next best method ranged from approximately 8% for the NR-ER-LBD dataset to as much as 27% for the SR-ARE and NR-Aromatase datasets. Understandably, the baseline classifier RF had the worst average performance even though its parameters were also optimized. SMN demonstrated a better F_1_ score in most cases because of its ability to improve recall without excessively lowering precision. A moderately higher recall value with comparable precision positively impacts the F_1_ score.

The Friedman’s Aligned Rank Test for Multiple Comparisons [55] was performed to further examine the statistical significance of the algorithmic effects of resampling techniques. Our null hypothesis was that all four algorithms had similar capability in classification measured by nine metrics for 12 datasets. Results shown in Table 4 suggest that all metrics except AUPRC were significantly affected by the resampling algorithm (*p* < 0.05). The Bergmann-Hommel post hoc analysis was applied to compare pairwise performance metrics of SMN against the other three classifiers. SMN differed more from RF than from SMO and RUS because one, two, and five metrics were insignificantly different (*p* > 0.05) between SMN and RF, SMN and SMO, and SMN and RUS, respectively. F_1_ score, MCC and Brier score showed significant difference among the four classifiers in both multiple and pair-wise comparisons. For instance, SMN had the lowest average Brier score of 0.3312 ± 0.0509 (average ± standard error) in comparison with SMO (0.4109 ± 0.0627), RUS (0.3894 ± 0.0361), and the baseline classifier RF (0.3967 ± 0.0395). A lower Brier score indicates that the predictions of a classifier are more accurate because they are closer to the ground truth. MCC, a metric widely used to evaluate the performance of SAR-based chemical classification [63, 64], embodies all the components of the confusion matrix and hence presents a reliable summary of the performance of models trained on imbalanced data.

**Table 4:** Friedman’s aligned rank test and Bergmann-Hommel post hoc analysis results showing corrected *p*-values for multiple and pair-wise comparisons between SMN and the other three classifiers, respectively

Comparisons	F_1_ score	AUPRC	AUROC	MCC	BA	Precision	Recall	Brier score	SSG
All four classifiers	0.0005	**0.1322**	0.0462	0.0111	5.4e−06	9.0e−05	1.8e−06	0.0017	2.0e−06
SMN vs RF	0.0003	**0.5253**	0.0168	0.0088	0.0001	0.0278	0.0013	0.0009	0.0010
SMN vs RUS	0.0051	**0.1008**	**0.0504**	0.0062	**1.0000**	**0.0948**	**0.2307**	0.0022	0.0274
SMN vs SMO	0.0003	**0.7818**	**0.3320**	0.0088	0.0001	0.0278	0.013	0.0007	8.4e−04

Insignificant statistics (*p* > 0.05) are highlighted in bold

On the contrary, AUPRC was the sole metric that did not differ significantly in any of the comparisons. AUPRC computes the area under the precision-recall curve that is obtained by using the output of the precision function at different recall levels to assess the overall performance of a prediction model [51]. SMN showed improved AUPRC scores compared to the other algorithms. However, this improvement was not very substantial. Unlike F_1_ score, which benefits from a varied classification threshold, minor improvements in the probabilities for each class do not translate to a marked improvement in the AUPRC score. This is because, being a threshold-independent metric, AUPRC computes the entire area under the curve for the plot of precision versus recall at all possible thresholds. Nevertheless, SMN still showed the best performance in 33% (4/12) of cases tested, RF and SMO in 25% (3/12) each, and RUS in 16% (2/12).

The above results suggest that AUPRC is not sensitive to algorithmic effects, whereas F_1_ score, MCC and Brier score are sensitive metrics that can distinguish among the classifiers by their performance. These results also indicate that SMN was the best performer, followed by RUS, while SMO and RF had the poorest performance with the Tox21 datasets. When looking at the average of all 9 metrics (Table 2), SMN and RUS ranked the best for 6 and 5 assays, separately, whereas RF only had the best performance with the NR-AR assay and SMO always underperformed across all 12 assays. These results led us to speculate that the activity landscape of the majority class (inactive compounds) may be more continuous and smooth than that of the minority class (active compounds) [65]. Consequently, removing some instances from the majority class would not affect class boundaries. On the contrary, adding synthetic instances to the minority class (SMOTE) may introduce noise along the borderlines, leading to the loss of activity cliffs and mislabeling of the synthetic instances [66]. The ENN algorithm may effectively remove those synthetic outliers and restore the activity cliffs and class boundaries, leading to enhanced prediction performance for SMOTEENN (SMN) [67]

### Comparison with Tox21 Data Challenge winners

In this section, we compared the prediction performance of the four classifiers in this study with those developed by the winning teams for each of the assays in the Tox21 Data Challenge [34]. The winning team for each sub-challenge was judged by AUROC (and BA if there was a tie in AUROC [35]). The AUROC and BA scores of the top ten ranked teams are posted at (https://tripod.nih.gov/tox21/challenge/leaderboard.jsp). The 12 assay sub-challenges were won by four teams: Bioinf@JKU, Amaziz, Dmlab and Microsomes. Bioinf@JKU developed DeepTox models using deep learning [25] and won six out of the 12 assay sub-challenges (NR-AhR, NR-AR-LBD, NR-ER, NR-PPAR-γ, SR-ARE, and SR-HSE) in addition to the Grand Challenge and two additional sub-challenges for the Nuclear Receptor Panel and the Stress Response Panel. Amaziz [68] employed associative neural networks to develop winning models for SR-ATAD5 and SR-MMP assays, and had the best overall BA score. Dmlab [58] used multi-tree ensemble methods, such as Random Forests and Extra Trees, to produce winning models for three assays (i.e., NR-AR, NR-aromatase and SR-p53). Microsomes [59] chose Random Forest for descriptor selection and model generation, and produced the best performing NR-ER-LBD model. For the purpose of comparison, we selected Dmlab and Microsomes because they used Random Forest. We also compared our best classifier with the winner of each assay sub-challenge. Given the over-optimistic nature of AUROC, the BA metric provides a more realistic and reliable measure for performance comparison. The titles of the best BA scores were shared by five teams: Kibutz (1 assay), Bioinf@JKU (2), Amaziz (2), T (3), and StructuralBioinformatics@Charite (4). The AUROC and BA scores of the winning teams are shown in Table 5 side by side with those of our best performing classifiers because they are the only metrics available for the Tox21 Data Challenge.

**Table 5:** Comparison between this study and Tox21 Data Challenge winners in terms of the classification performance metrics AUROC and balanced accuracy

Assay ID	AUROC					Balanced accuracy (BA)					Best classifier / challenge winner	
	Best classifier (this study)		Dmlab [58]	Microsomes [59]	Challenge winner	Best classifier (this study)		Dmlab [58]	Microsomes [59]	Challenge winner		
	Value	Name				Value	Name				AUROC	BA
NR-AR	0.823	RF	**0.830**	N/A	0.828	0.644	SMN	0.610	N/A	0.736	0.994	0.875
NR-AR-LBD	*0.913*	RUS	0.820	N/A	0.879	0.612	RUS	0.490	N/A	0.650	1.039	0.942
NR-AhR	0.920	SMN	0.780	0.901	0.928	0.823	SMN	0.560	0.698	0.853	0.991	0.965
NR-Aromatase	*0.850*	SMN	**0.840**	N/A	0.838	0.727	SMN	0.560	N/A	0.737	1.014	0.986
NR-ER	*0.863*	SMN	0.770	0.783	0.810	*0.792*	SMN	0.660	0.621	0.749	1.065	1.057
NR-ER-LBD	0.823	SMN	0.770	**0.827**	0.827	0.697	RUS	0.590	0.550	0.715	0.995	0.975
NR-PPAR-γ	0.794	RUS	0.830	0.718	0.861	0.745	RUS	0.550	N/A	0.785	0.922	0.949
SR-ARE	*0.891*	SMN	0.770	0.804	0.840	*0.855*	SMN	0.520	0.605	0.729	1.061	1.173
SR-ATAD5	0.815	SMO	0.800	0.812	0.828	0.713	RUS	0.610	0.539	0.741	0.984	0.962
SR-HSE	0.848	SMN	0.860	N/A	0.865	0.667	RUS	0.560	N/A	0.799	0.980	0.835
SR-MMP	0.930	RUS	0.950	N/A	0.950	0.852	RUS	0.69	N/A	0.904	0.978	0.942
SR-p53	0.879	SMN	**0.880**	0.826	0.880	*0.778*	RUS	0.58	0.523	0.765	0.998	1.017
Average	*0.862*		0.830	0.810	0.861	0.742		0.58	0.589	0.764	1.002	0.973

The values in italics are the highest among all the classifiers (both this study and Tox21 Data Challenge) whereas the values in bold font are the best among the Tox21 Data Challenge participating teams [34]

Although the AUROC and BA metrics are not ideal for evaluating imbalanced classification, we made the comparison to demonstrate that the improvement obtained from imbalance pre-processing enabled our classifiers to perform equally well or outperform the winning models of the Tox21 Data Challenge. This is primarily reflected by the following observations: (1) our best classifiers outperformed Dmlab and Microsomes in terms of both AUROC and BA by large margins with only four exceptions (NR-AR, NR-PPAR-γ, SR-ATAD5 and SR-MMP), where Dmlab exceeded our best classifiers in AUROC by less than 4%; (2) our best classifiers had the same or higher AUROC and a higher BA than challenge winners for six and three assays, respectively, with less than 8% (AUROC) or 17% (BA) difference for the remaining assays; and (3) on average, our best classifiers performed almost equally as well as the challenge winners as a whole (Table 5). The last two columns in Table 5 report the comparison between our best classifier and the winner of Tox21 Challenge in terms of BA and AUROC ratios, with a value greater than 1 indicating that our model performed better than the Challenge winning model. These results (particularly the BA scores) not only establish the validity, credibility and scientific soundness of the approach, methodology and algorithms implemented in this study, but also demonstrate that the excellence of our work reached levels comparable to that of the Tox21 Data Challenge winners.

It is also worth noting that Banerjee et al. [13] performed similar work on three Tox21 datasets (AhR, ER-LDB, and HSE). They employed RF as the base classifier (without ensemble learning) and applied eight different undersampling or oversampling techniques (including random undersampling and SMOTE). Similar to this study, their work also demonstrated that dataset and resampling techniques had significant impacts on classification outcome and that such impacts varied from one metric to another with sensitivity and F-measure being more sensitive than AUROC and accuracy.

Another study worth mentioning described how Norinder and Boyer [16] achieved balanced prediction performance with sensitivity and specificity (for the external test dataset) both attaining 0.70 − 0.75 when they applied MCP to the similar ToxCast and Tox21 datasets of estrogen receptor assays and used SVM as the classifier. These results are far superior to those obtained using SVM or RF alone without resampling or MCP [16, 69], but they are only slightly better than the performance of RUS with sensitivity at 0.69 or 0.55 (Table 2) and specificity at 0.61 or 0.84 (Additional file 1: Table S1) obtained in our study. Therefore, it warrants further in-depth investigations to compare side-by-side resampling with MCP and MCP + resampling using the same machine learning algorithms, the same raw datasets, and the same preprocessing procedure.

## Conclusions

Due to the specificity of toxicant-target biomolecule interactions, SAR-based chemical classification studies are often impeded by the imbalanced nature of many toxicity datasets. Furthermore, class boundaries are often blurred since active toxicants often appear in the minority class. In order to address these issues, common resampling techniques can be applied. However, removing majority class instances using an undersampling technique can result in information loss, whereas increasing minority instances by interpolation tends to further obfuscate the majority class space, giving rise to over-fitting. In order to improve the prediction accuracy attained from imbalanced learning, SMOTEENN, a combination of SMOTE and ENN algorithms, is often employed to oversample the minority class by creating synthetic samples, followed by cleaning the mislabeled instances. Here, we integrated an ensemble approach (bagging) with a base classifier (RF) and various resampling techniques to form four learning algorithms (RF, RUS, SMO and SMN). Then, we applied them to the binary classification of 12 highly imbalanced Tox21 in vitro qHTS bioassay datasets.

We generated multiple sets of chemical descriptors or fingerprints and down-selected small groups of features for use in class prediction model generation. After data preprocessing, parameters were optimized for both resampling and classifier training. The performance of the four learning methods was compared using nine evaluation metrics, among which F_1_ score, MCC and Brier score provided more consistent assessment of the overall performance across the 12 datasets. The Friedman’s aligned ranks test and the subsequent Bergmann-Hommel post hoc test showed that SMN significantly outperformed the other three methods. It was also found that there was a strong negative correlation between prediction accuracy and IR. We observed that SMN became less effective when IR exceeded a certain threshold (e.g., > 28). Therefore, SAR-based imbalanced learning can be affected by the degree of dataset skewness, resampling algorithms, and evaluation metrics. We recommend assembling a panel of representative, diversified and imbalance-sensitive metrics, developing a comprehensive index from this panel, and using the index to evaluate the performance of classifiers for imbalanced datasets.

The ability to separate the small number of active compounds from the vast amounts of inactive ones is of great importance in computational toxicology. This work demonstrates that the performance of SAR-based, imbalanced chemical toxicity classification can be significantly improved through imbalance handling. Although the best classifiers of this study achieved the same level of performance as the winners of the Tox21 Data Challenge as a whole, we believe that there is still plenty of room for further improvement. Given the exceptionally outstanding performance of DeepTox [25] and our own experience with deep learning-based chemical toxicity classification [70], our future plan is to replace RF with a deep learning algorithm like deep neural networks as the base classifier and combine it with class rebalancing techniques to build novel deep learning models for SAR-based chemical toxicity prediction. We are also interested in pursuing a novel approach by integrating MCP, resampling and ensemble strategies to further improve the robustness and performance of imbalanced learning.

## Supplementary information

**Additional file 1: Text S1.** SMOTEENN algorithm. **Figure S1.** Illustration of SMOTE and ENN techniques. (a) The original imbalanced data; (b) Synthetic samples are generated for the minority class using SMOTE. (c) Using ENN, those mislabeled synthetic samples were removed from the minority class. (d) The rebalanced data after the application of SMOTEENN. **Table S1.** Evaluation metrics derived for four classification methods (RF, RUS, SMO and SMN) with twelve Tox21 qHTS assay datasets. Specificity and two other metrics (sensitivity and SSG, both appearing in Table 2) are shown.

## Data Availability

The dataset supporting the conclusions of this article is available at https://tripod.nih.gov/tox21/challenge/data.jsp in sdf and smi formats. The source code of this article is available at https://github.com/Idakwo/SAR_Imbalance_SMOTEENN.

## Abbreviations

ANOVA: Analysis of variance
AUPRC: Area Under the Precision-Recall Curve
AUROC: Area Under the Receiver Operating Characteristics
BA: Balanced accuracy
CART: Classification and Regression Trees (Decision trees)
CC: Correlation coefficient
CV: Coefficient of variation
df: Degree of freedom
ENN: Edited Nearest Neighbor (ENN) algorithm
FN: False negative
FP: False positive
IR: Imbalance ratio
KNN: K-nearest neighbors
MCC: Matthews correlation coefficient
MLP: Multilayer perceptron
NB: Naïve Bayes
RF: Random Forest classification method
RUS: Random Undersampling
SAR: Structure–Activity Relationship
SMN: RF classification method with SMOTEENN technique
SMO: RF classification method with SMOTE technique
SMOTE: Synthetic Minority Over-sampling Technique (SMOTE)
SMOTEENN: Combined the SMOTE technique with the ENN algorithm
SVM: Support vector machine
TN: True negative
TNR: True negative rate
Tox21: Toxicology in the 21st Century program
TP: True positive
TPR: True positive rate

## References

[1] Czarnecki WM, Rataj K (2015). Compounds activity prediction in large imbalanced datasets with substructural relations fingerprint and EEM. 2015 IEEE Trustcom/BigDataSE/ISPA.

[2] Irwin JJ, Sterling T, Mysinger MM (2012). ZINC: a free tool to discover chemistry for biology. J ChemInf Model.

[3] Dahl GE, Jaitly N, Salakhutdinov R (2014) Multi-task neural networks for QSAR predictions. https://arxiv.org/abs/1406.1231. Accessed 6 Oct 2017

[4] Darnag R, Mostapha Mazouz EL, Schmitzer A (2010). Support vector machines: development of QSAR models for predicting anti-HIV-1 activity of TIBO derivatives. Eur J Med Chem.

[5] Polishchuk PG, Muratov EN, Artemenko AG (2009). Application of random forest approach to QSAR prediction of aquatic toxicity. J ChemInf Model.

[6] Galar M, Fernández A, Barrenechea E (2012). A review on ensembles for the class imbalance problem: bagging-, boosting-, and hybrid-based approaches. IEEE Trans Syst Man Cybern Part C.

[7] Krawczyk B, Krawczyk BB (2016). Learning from imbalanced data: open challenges and future directions. Prog Artif Intell.

[8] Hido S, Kashima H, Takahashi Y (2009). Roughly balanced bagging for imbalanced data. Stat Anal Data Min.

[9] Chawla NV, Maimon O, Rokach L (2005). Data mining for imbalanced datasets: an overview. Data Mining and Knowledge Discovery Handbook.

[10] He H, Ma Y (2013). Imbalanced learning: foundations, algorithms, and applications.

[11] Branco P, Torgo L, Ribeiro R (2015) A survey of predictive modelling under imbalanced distributions. https://arxiv.org/abs/1505.01658. Accessed 8 Aug 2017

[12] Chawla NV, Bowyer KW, Hall LO, Kegelmeyer WP (2002). SMOTE: Synthetic Minority Over-sampling Technique. J Artif Intell Res.

[13] Banerjee P, Dehnbostel FO, Preissner R (2018). Prediction is a balancing act: importance of sampling methods to balance sensitivity and specificity of predictive models based on imbalanced chemical data sets. Front Chem.

[14] Stefanowski J (2016). Dealing with Data Difficulty Factors While Learning from Imbalanced Data. Challenges in computational statistics and data mining.

[15] Bosc N, Atkinson F, Felix E (2019). Large scale comparison of QSAR and conformal prediction methods and their applications in drug discovery. J Cheminform.

[16] Norinder U, Boyer S (2016). Conformal Prediction Classification of a Large Data Set of EnRvironmental Chemicals from ToxCast and Tox21 Estrogen Receptor Assays. Chem Res Toxicol.

[17] Sun J, Carlsson L, Ahlberg E (2017). Applying mondrian cross-conformal prediction to estimate prediction confidence on large imbalanced bioactivity data sets. J ChemInf Model.

[18] Cortés-Ciriano I, Bender A (2019) Concepts and applications of conformal prediction in computational drug discovery

[19] Norinder U, Boyer S (2017). Binary classification of imbalanced datasets using conformal prediction. J Mol Graph Model.

[20] He H, Garcia EA (2009). Learning from Imbalanced Data. IEEE Trans Knowl Data Eng.

[21] Davis J, Goadrich M (2006) The relationship between precision-recall and ROC curves. In: Proceedings of the 23rd International Conference on Machine Learning. ACM, Pittsburgh, pp 233–240

[22] Provost F, Fawcett T, Kohavi R (1998) The case against accuracy estimation for comparing induction algorithms. In: Proceedings of the Fifteenth International Conference on Machine Learning. Morgan Kaufmann Publishers Inc, San Francisco, pp 445–453

[23] Capuzzi SJ, Politi R, Isayev O (2016). QSAR modeling of Tox21 challenge stress response and nuclear receptor signaling toxicity assays. Front Environ Sci.

[24] Ribay K, Kim MT, Wang W (2016). Predictive modeling of estrogen receptor binding agents using advanced cheminformatics tools and massive public data. Front Environ Sci.

[25] Mayr A, Klambauer G, Unterthiner T (2016). DeepTox: toxicity prediction using deep learning. Front Environ Sci.

[26] Drwal MN, Siramshetty VB, Banerjee P (2015). Molecular similarity-based predictions of the Tox21 screening outcome. Front Environ Sci.

[27] Saito T, Rehmsmeier M, Hood L (2015). The precision-recall plot is more informative than the ROC plot when evaluating binary classifiers on imbalanced datasets. PLoS ONE.

[28] Chen J, Tang YY, Fang B, Guo C (2012). In silico prediction of toxic action mechanisms of phenols for imbalanced data with Random Forest learner. J Mol Graph Model.

[29] Pham-The H, Casañola-Martin G, Garrigues T (2016). Exploring different strategies for imbalanced ADME data problem: case study on Caco-2 permeability modeling. Mol Divers.

[30] Lei T, Sun H, Kang Y (2017). ADMET evaluation in drug discovery. 18. Reliable prediction of chemical-induced urinary tract toxicity by boosting machine learning approaches. Mol Pharm.

[31] Czarnecki WM, Tabor J (2017). Extreme entropy machines: robust information theoretic classification. Pattern Anal Appl.

[32] Batista GEAPA, Prati RC, Monard MC (2004). A study of the behavior of several methods for balancing machine learning training data. ACM SIGKDDExplorNewsl.

[33] NCATS Toxicology in the 21st Century (Tox21). https://ncats.nih.gov/tox21. Accessed 11 May 2017

[34] Huang R, Xia M, Nguyen D-T (2016). Editorial: Tox21 challenge to build predictive models of nuclear receptor and stress response pathways as mediated by exposure to environmental toxicants and drugs. Front Environ Sci.

[35] Huang R, Xia M, Nguyen D-T (2017). Tox21Challenge to build predictive models of nuclear receptor and stress response pathways as mediated by exposure to environmental chemicals and drugs.

[36] MolVS: Molecule Validation and Standardization—MolVS 0.0.9 documentation. https://molvs.readthedocs.io/en/latest/. Accessed 6 Feb 2018

[37] Greg L RDKit: Open-source cheminformatics Software

[38] Tropsha A, Gramatica P, Gombar V (2003). The importance of being Earnest: validation is the absolute essential for successful application and interpretation of QSPR models. QSAR Comb Sci.

[39] Stefaniak F (2015). Prediction of compounds activity in nuclear receptor signaling and stress pathway assays using machine learning algorithms and low-dimensional molecular descriptors. Front Environ Sci.

[40] Rogers D, Hahn M (2010). Extended-connectivity fingerprints. J ChemInf Model.

[41] Seiffert C, Khoshgoftaar TM, Van Hulse J, Napolitano A (2010). RUSBoost: a hybrid approach to alleviating class imbalance. IEEE Trans Syst Man, Cybern Part ASyst Humans.

[42] García V, Sánchez JS, Mollineda RA (2012). On the effectiveness of preprocessing methods when dealing with different levels of class imbalance. Knowl Based Syst.

[43] Galar M, Fernández A, Barrenechea E, Herrera F (2013). EUSBoost: enhancing ensembles for highly imbalanced data-sets by evolutionary undersampling. Pattern Recognit.

[44] Wilson DL (1972) Asymptotic Properties of Nearest Neighbor Rules Using Edited Data. IEEE Trans Syst Man Cybern 3:408–421. doi.:10.1109/TSMC.1972.4309137

[45] Breiman L (2001). Random forests. Mach Learn.

[46] Han J, Kamber M, Pei J (2011). Data mining : concepts and techniques.

[47] Altman N, Krzywinski M (2017). Ensemble methods: bagging and random forests. Nat Methods.

[48] Khoshgoftaar TM, Van Hulse J, Napolitano A (2011). Comparing boosting and bagging techniques with noisy and imbalanced data. IEEE Trans Syst Man Cybern Part A Syst Humans.

[49] Laszczyski J, Stefanowski J, Idkowiak L (2013) Extending bagging for imbalanced data. In: Burduk R., Jackowski K., Kurzynski M., Wozniak M., Zolnierek A. (eds) Proceedings of the 8th International Conference on Computer Recognition Systems CORES 2013. Advances in Intelligent Systems and Computing. Springer, Heidelberg, pp 269–278

[50] Chawla NV, Lazarevic A, Hall LO, Bowyer KW (2003). SMOTEBoost: improving prediction of the minority class in boosting.

[51] Pedregosa F, Varoquaux G, Gramfort A (2011). Scikit-learn: machine learning in python. J Mach Learn Res.

[52] Lemaˆıtre G, Nogueira F, Aridas CK (2017). Imbalanced-learn: a python toolbox to tackle the curse of imbalanced datasets in machine learning. J Mach Learn Res.

[53] Boughorbel S, Jarray F, El-Anbari M (2017). Optimal classifier for imbalanced data using Matthews Correlation Coefficient metric. PLoS ONE.

[54] Bergmann B, Hommel G (1988). Improvements of general multiple test procedures for redundant systems of hypotheses.

[55] García S, Fernández A, Luengo J, Herrera F (2010). Advanced nonparametric tests for multiple comparisons in the design of experiments in computational intelligence and data mining: experimental analysis of power. InfSci (Ny).

[56] Calvo B, Santafé G (2016). scmamp: Statistical comparison of multiple algorithms in multiple problems. R J.

[57] Hodges JL, Lehmann EL, Rojo J (2012). Rank methods for combination of independent experiments in analysis of variance. Selected works of E L. Lehmann.

[58] Barta G (2016). Identifying biological pathway interrupting toxins using multi-tree ensembles. Front Environ Sci.

[59] Uesawa Y (2016). Rigorous selection of random forest models for identifying compounds that activate toxicity-related pathways. Front Environ Sci.

[60] Kuncheva LI, Whitaker CJ (2003). Measures of diversity in classifier ensembles and their relationship with the ensemble accuracy. Mach Learn.

[61] Ferri C, Hernández-Orallo J, Modroiu R (2009). An experimental comparison of performance measures for classification. Pattern Recognit Lett.

[62] Jeni LA, Cohn JF, De La Torre F (2013) Facing imbalanced data—recommendations for the use of performance metrics. In: 2013 Humaine Association Conference on Affective Computing and Intelligent Interaction. IEEE, New York, pp 245–251 10.1109/ACII.2013.47 PMC4285355 25574450

[63] Tong W, Hong H, Fang H (2003). Decision forest: combining the predictions of multiple independent decision tree models. J ChemInfComputSci.

[64] Sakkiah S, Selvaraj C, Gong P (2017). Development of estrogen receptor beta binding prediction model using large sets of chemicals. Oncotarget.

[65] Cruz-Monteagudo M, Medina-Franco JL, Pé Rez-Castillo Y (2014). Activity cliffs in drug discovery: Dr Jekyll or Mr Hyde?. Drug Discov Today.

[66] Stumpfe D, Hu H, Bajorath J (2019). Evolving concept of activity cliffs. ACS Omega.

[67] Yang Z, Gao D (2013). Classification for imbalanced and overlapping classes using outlier detection and sampling techniques.

[68] Abdelaziz A, Spahn-Langguth H, Schramm K-W, Tetko IV (2016). Consensus modeling for HTS assays using in silico descriptors calculates the best balanced accuracy in Tox21 challenge. Front Environ Sci.

[69] Zang Q, Rotroff DM, Judson RS (2013). Binary classification of a large collection of environmental chemicals from estrogen receptor assays by quantitative structure-activity relationship and machine learning methods. J Chem Inf Model.

[70] Idakwo G, Thangapandian S, Luttrell J (2019). Deep learning-based structure-activity relationship modeling for multi-category toxicity classification: a case study of 10KTox21 chemicals with high-throughput cell-based androgen receptor bioassay data. Front Physiol.

