# Supplementary material for: Structure–activity relationship-based chemical classification of highly imbalanced Tox21 datasets
Source: J Cheminform. 2020 Oct 27;12:66. doi: 10.1186/s13321-020-00468-x (PMC7592558; doi:10.1186/s13321-020-00468-x)
Supplement: Supplementary file 1 — Additional file 1: Text S1. SMOTEENN algorithm. Figure S1. Illustration of SMOTE and ENN techniques. (a) The original imbalanced data; (b) Synthetic samples are generated for the minority class using SMOTE. (c) Using ENN, those mislabeled synthetic samples were removed from the minority class. (d) The rebalanced data after the application of SMOTEENN. Table S1. Evaluation metrics derived for four classification methods (RF, RUS, SMO and SMN) with twelve Tox21 qHTS assay datasets. Specificity and two other metrics (sensitivity and SSG, both appearing in Table 2) are shown. [file 13321_2020_468_MOESM1_ESM.docx]

**SUPPLEMENTARY MATERIALS**

**Structure-activity relationship-based chemical classification of highly imbalanced Tox21 datasets**

Gabriel Idakwo^1^, Sundar Thangapandian^2^, Joseph Luttrell^1^, Yan Li^3^, Nan Wang^4^, Zhaoxian Zhou^1^, Huixiao Hong^5^, Bei Yang^6^, Chaoyang Zhang^1*^, Ping Gong^2*^

**Text S1: SMOTEENN algorithm**

Illustrated below is an explanation of how the combination of SMOTE and ENN creates new synthetic samples for the minority class using SMOTE and then cleans the mislabeled synthetic samples using ENN.

Consider $D$ as the training data. $D_{min}$ represents samples of the minority class, denoted by the red color samples in Figure S1(a). For every sample $p_{1},$ $p_{1} \in D_{min}$, find $k$ nearest neighbors of $p_{1}$. Compute the vector difference $d$ between $p_{1}$ and a selected nearest neighbor $p_{2}$obtained based on Euclidean distance. Next, compute the product of $d$ and $\alpha$ where $\alpha\in(0,1)$. The sum of the product and $p_{1}$ is the new synthetic minority sample, $p_{new}$. As a result, $p_{new}$ will appear as a point between $p_{1}$ and $p_{2}$, as shown in Figure S1(b). Similarly, we can create another synthetic sample $q_{new}$using SMOTE. Both $p_{new} \mathrm{and}q_{new}$have the minority class label (active) because they are created using minority samples. However, most neighbors of $q_{new}$ belong to the majority class with an inactive label so this synthetic sample is mislabeled and must be removed, while $p_{new}$ is retained since its label is consistent with the label of most of its neighbors, as shown in Figure S1(b) and Figure S1(c). ENN involves using *k*-nearest neighbors to locate those examples in a dataset that are misclassified and that are then removed (Figure S1(c)). ENN ensures that a valid synthetic sample is located in the same feature space as the original minority class, thus reducing the chance of introducing new outliers. SMOTEENN enables us to augment the minority class (Figure S1(d)) and obtain a well-balanced training dataset, which may help improve the performance of SAR-based chemical classification.


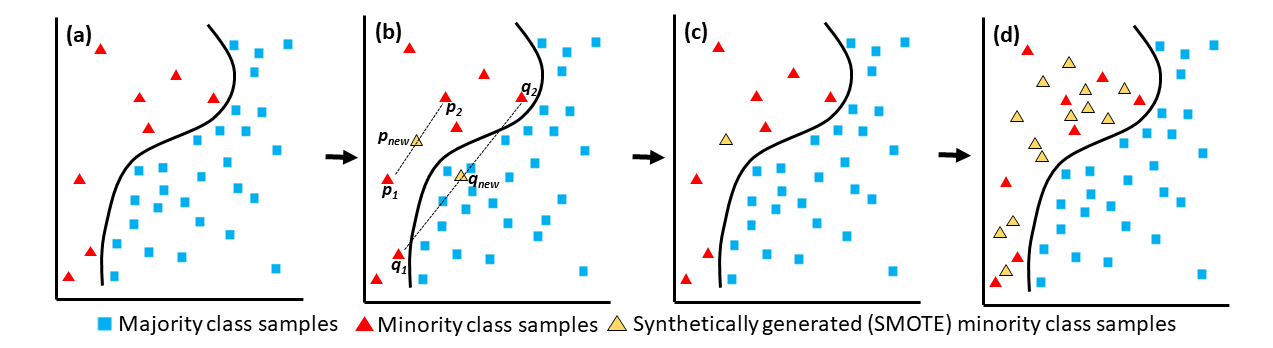


**Figure S1.** Illustration of SMOTE and ENN techniques. (a) The original imbalanced data; (b) Synthetic samples are generated for the minority class using SMOTE. (c) Using ENN, those mislabeled synthetic samples were removed from the minority class. (d) The rebalanced data after the application of SMOTEENN.

**Table S1**. Evaluation metrics derived for four classification methods (RF, RUS, SMO and SMN) with twelve Tox21 qHTS assay datasets. Specificity and two other metrics (sensitivity and SSG, both appearing in Table 2) are shown.

| **Metrics** | **Classifier** | **NR-AR** | **NR-AR-LBD** | **NR-AhR** | **NR-Aromatase** | **NR-ER** | **NR-ER-LBD** | **NR-PPAR-γ** | **SR-ARE** | **SR-ATAD5** | **SR-HSE** | **SR-MMP** | **SR-p53** | **Mean** | **CV** |
| --- | --- | --- | --- | --- | --- | --- | --- | --- | --- | --- | --- | --- | --- | --- | --- |
| **Specificity** | **RF** | 1.0000 | 0.9982 | 0.9750 | 0.9979 | 0.9824 | 0.9964 | 0.9946 | 0.9531 | 0.9982 | 0.9930 | 0.9661 | 0.9946 | 0.9875 | 2% |
|  | **RUS** | 0.9357 | 0.9749 | 0.7115 | 0.6088 | 0.6088 | 0.8437 | 0.9391 | 0.6228 | 0.6620 | 0.6859 | 0.8898 | 0.6344 | 0.7598 | 19% |
|  | **SMO** | 0.9964 | 0.9964 | 0.9750 | 0.9979 | 0.9912 | 0.9929 | 0.9964 | 0.9621 | 0.9965 | 0.9965 | 0.9746 | 0.9964 | 0.9894 | 1% |
|  | **SMN** | 0.9554 | 0.9838 | 0.8885 | 0.9665 | 0.9516 | 0.9716 | 0.9713 | 0.8795 | 0.9331 | 0.9529 | 0.8941 | 0.9677 | 0.9430 | 4% |
| **Sensitivity** | **RF** | 0.0833 | 0.0000 | 0.3286 | 0.1351 | 0.1837 | 0.1500 | 0.0345 | 0.2500 | 0.1471 | 0.1765 | 0.4444 | 0.0789 | 0.1677 | 75% |
|  | **RUS** | 0.2500 | 0.2500 | 0.9143 | 0.7568 | 0.6939 | 0.5500 | 0.5517 | 0.7727 | 0.7647 | 0.6471 | 0.8148 | 0.9211 | 0.6573 | 34% |
|  | **SMO** | 0.1667 | 0.0000 | 0.2857 | 0.1081 | 0.2449 | 0.2000 | 0.1034 | 0.2045 | 0.1471 | 0.1176 | 0.2963 | 0.0789 | 0.1628 | 54% |
|  | **SMN** | 0.3333 | 0.1250 | 0.7571 | 0.4865 | 0.6327 | 0.4000 | 0.3793 | 0.8295 | 0.4706 | 0.3529 | 0.7963 | 0.3947 | 0.4965 | 43% |
| **Sensitivity-Specificity Gap (SSG)** | **RF** | 0.9167 | 0.9982 | 0.6464 | 0.8628 | 0.7987 | 0.8464 | 0.9601 | 0.7031 | 0.8511 | 0.8165 | 0.5217 | 0.9157 | 0.8198 | 17% |
|  | **RUS** | 0.6857 | 0.7249 | 0.2028 | 0.1480 | 0.0851 | 0.2937 | 0.3874 | 0.1499 | 0.1027 | 0.0388 | 0.0750 | 0.2867 | 0.2651 | 87% |
|  | **SMO** | 0.8297 | 0.9964 | 0.6893 | 0.8898 | 0.7463 | 0.7929 | 0.8930 | 0.7576 | 0.8494 | 0.8789 | 0.6783 | 0.9175 | 0.8266 | 12% |
|  | **SMN** | 0.6221 | 0.8588 | 0.1314 | 0.4800 | 0.3189 | 0.5716 | 0.5920 | 0.0500 | 0.4625 | 0.6000 | 0.0978 | 0.5730 | 0.4465 | 55% |
